# Extracellular vesicles in seminal plasma of *Sahiwal* cattle bulls carry a differential abundance of sperm fertility-associated proteins for augmenting the functional quality of low-fertile bull spermatozoa

**DOI:** 10.1038/s41598-025-87998-2

**Published:** 2025-01-28

**Authors:** Ankit Pal, Seema Karanwal, Mir Ahmad Habib, Fanny Josan, Vikrant Gaur, Aditya Patel, Muskaan Garg, Mukesh Bhakat, Tirtha K. Datta, Rakesh Kumar

**Affiliations:** 1 Animal Genomics Laboratory, Animal Biotechnology Division, ICAR-National Dairy Research Institute, Karnal, Haryana India; 2 Molecular Biology Unit, Dairy Microbiology Division, ICAR-National Dairy Research Institute, Karnal, Haryana India; 3 Artificial Breeding Research Centre, LPM Division, ICAR-National Dairy Research Institute, Karnal, Haryana India; 4 ICAR-Central Institute for Research on Buffaloes, Hisar, Haryana India

**Keywords:** Seminal plasma, Extracellular vesicles, Spermatozoa, Protein, *Sahiwal* bulls, High fertile, Low fertile, Biochemistry, Biotechnology, Cell biology, Developmental biology

## Abstract

Poor male fertility significantly affects dairy production, primarily due to low conception rates (CR) in bulls, even when cows are inseminated with morphologically normal sperm. Seminal plasma is a key factor in evaluating the fertilizing ability of bull semen. The extracellular vesicles (EVs) in seminal plasma contain fertility-associated proteins like SPAM1, ADAM7, and SP10, which influence sperm function and fertilizing potential. This study aimed to assess EV-associated fertility proteins in bulls with varying fertility levels and to investigate the effects of seminal plasma EVs (SPEVs) from high-fertility (HF) bulls on the spermatozoa of low-fertility (LF) *Sahiwal* bulls. Seminal plasma was isolated from the fresh semen of *Sahiwal* bulls, with four bulls classified as high fertility and three as low fertility. Fertility-associated proteins such as SP10, ADAM7, and SPAM 1 were highly expressed in SPEVs of high-fertility (HF) bulls. PKH26 dye-labeled SPEVs demonstrated significant uptake by spermatozoa at pH 6.8 with ≥ 4 h of co-incubation. Exposure of HF SPEVs to low-fertility (LF) spermatozoa reduced acrosome response, capacitation, and reactive oxygen species (ROS) formation. Our findings suggest that protein repertoires in SPEVs influence sperm activities such as motility, acrosome response, and ROS production. Supplementing LF spermatozoa with HF SPEVs could enhance their functional characteristics, highlighting these proteins as potential resources to modulate cattle bull sperm fertilizing ability.

**Supplementary Information:**

The online version contains supplementary material available at 10.1038/s41598-025-87998-2.

## Introduction

As the global population grows and economies expand, the demand for livestock products is on the rise, especially in developing countries. The livestock sector can contribute by promoting economic growth, social development, and sustainable resource management. In these regions, livestock serves as a major asset, livelihood sector, and a source of high-quality nutrients. However, livestock consistently faces reproductive challenges like longer calving intervals, low conception rates, and early embryonic loss. These issues reduce the reproductive efficiency of dairy animals, leading to financial losses in the dairy industry^1,2^. Dairy cattle bulls exhibit considerable variability in their ability to give full-term pregnancies, with certain breeds renowned for their superior milking capabilities, such as the *Sahiwal* breed in India^3,4^. As per studies, infertility is prevalent in both males and females, however, its impact on a male is particularly significant since semen from a single bull is utilized for artificial breeding across thousands of cows^5^. While female fertility has been extensively addressed and improved through assisted reproductive technologies and genetic selection, bull fertility has unfortunately been overlooked^3^. Bull subfertility attributed to inadequate semen quality, contributes significantly to global reproductive failures in livestock^6^.

In addition to the cellular components of semen, the seminal plasma constitutes 95% of the ejaculate and plays a crucial role in male fertility. The seminal plasma is the non-cellular liquid consisting of secretion from the testes, epididymes, bulbourethral glands, prostate, and the ampullae of the ductus deferens, with a major contribution of the seminal vesicles^7^**.** Moreover**,** seminal plasma is enriched with proteins, nucleic acid, and lipids encapsulated within the seminal plasma extracellular vesicles (SPEVs) or exosomes^8^. In addition, seminal plasma contains a heterogeneous population of extracellular vesicles (EVs) produced and released by the organs in the male genital tract including the prostate, Epididymes, and other accessory genital glands^9^. The released EVs promote intercellular communication by transferring their cargo to the recipient cells.

In the male reproductive system, spermatozoa are encountered by EVs, which regulate their metabolism and composition through intercellular communication^10^. Earlier studies have reported that SPEVs play a major role in affecting human sperm function^11^. These SPEVs provide immunosuppressive and antibacterial protection for spermatozoa. As the spermatozoa traverse the male reproductive tract, specific components, such as epididymosomes and prostasomes, contribute to imparting a distinct identity to them^10,12^. This interaction endows them with the ability to progress and fertilize an egg. Seminal plasma vesicles exercise regulatory control over key sperm functions, including motility, the acrosome reaction, capacitation^13–15^. Existing research demonstrates the involvement of SPEVs in affecting the cells of the female reproductive tract to promote successful reproduction and help sperm reach functional maturity^16^. Additionally, SPEVs have demonstrated involvement in reducing oxidative stress associated with cryopreservation^17^.

A noteworthy observation is that adding boar seminal plasma exosomes into semen enhances sperm motility, prolongs effective survival time, improves sperm plasma membrane integrity, prevents premature capacitation, and elevates total antioxidant capacity (T-AOC)^18^. It has been reported that SPEVs may aid in the removal and addition of proteins to the ram sperm membrane^19^. This implies that specific proteins may play a pivotal role in regulating the functions of spermatozoa. Additionally, SPEVs derived from normozoospermic men enhance sperm motility and induce capacitation in vasectomized men^20^. SPEVs of high-fertile (HF) bulls when incubated with the sperm of low-fertile (LF) bulls improve their fertilizing capacity^21^. Bull seminal plasma contains a variety of fertility factors and determinants, including SP10, ADAM7, SPAM1, and a diverse protein repertoire^22–24^. During fertilization, SPAM1 allows acrosome-intact sperm to penetrate the cumulus cell layer surrounding the oocyte, due to the insoluble hyaluronidase activity at neutral pH, and is required for acrosome-reacted sperm to bind to the zona pellucida (ZP)^25^. A transmembrane protein ‘a disintegrin and metalloproteinase 7’ (ADAM7) gets transferred by epididymosomes to the sperm cells and plays a role in sperm-oocyte interaction^26^. SP10, a sperm intra-acrosomal protein, unique to the testis, plays a crucial role in egg-sperm binding^27^. Considering these findings, it is logical that the cargo SPEVs may impact semen fertility, suggesting that SPEVs obtained from HF bulls could potentially enhance the semen quality of less fertile counterparts. To test this hypothesis, initially, SPEVs from the seminal plasma of *Sahiwal* bulls of proven fertility were isolated and incorporated into the sperm of LF bulls at different time intervals and pH. Further, the relative abundance of fertility-related protein was determined in the HF and LF bulls, and the effectiveness of SPEVs in improving the semen quality of bulls of proven low fertility was evaluated using various sperm function parameters.

## Results

### Isolation of SPEVs and characterization

**Fig. 1:**
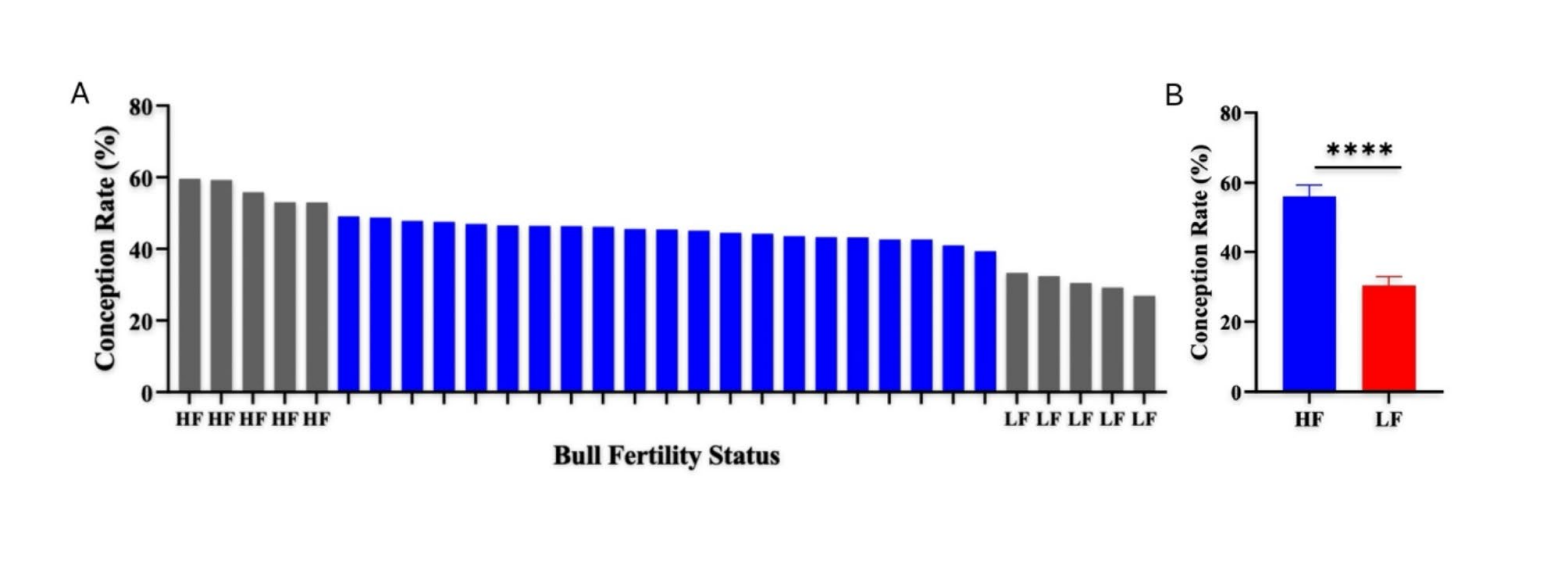
Selection of HF and LF *Sahiwal* cattle bulls based on conception rate (CR). (**A**) Five bulls with the highest CRs were considered HF while five bulls with the lowest CRs were considered as LF. (**B**) The mean CRs of shortlisted HF and LF bulls were significantly different (p < 0.0001).

Analysis using dynamic light scattering (DLS) revealed the presence of heterogeneous large particles in the initial size exclusion chromatography (SEC) fractions (1–6), while the average size of EVs decreased in the later fractions. Notably, fractions 7–12 contained particles with sizes within the expected range of EVs (70 – 200 nm) and showed a uniform particle distribution. The average particle size in fractions 1–6 was found to be more than 350 nm with a heterogenous population of particles (Fig. 1A, Table 1). Fractions 7–12 showed homogeneity of EV population with a zeta size average from 189 to 159 nm, classified EV population size. Fraction 13 onwards showed a heterogeneous population of nanoparticles with decreasing zeta average size.

**Table 1:** Intensity-based size distribution of seminal plasma-derived EVs, as assessed by the Zetasizer nano zs particle sizer in size exclusion chromatography fractions.

S. No	SEC fraction no	Average particle size	Polydispersity index (PDI) value
1	1–2	1672	0.031
2	3–4	1319	0.934
3	5–6	349.1	0.668
4	7–8	189.1	0.234
5	9–10	175.4	0.265
6	11–12	159.3	0.435
7	13–14	176.0	0.436
8	15–16	139.4	0.464
9	17–18	341.4	0.789

**Fig. 2:**
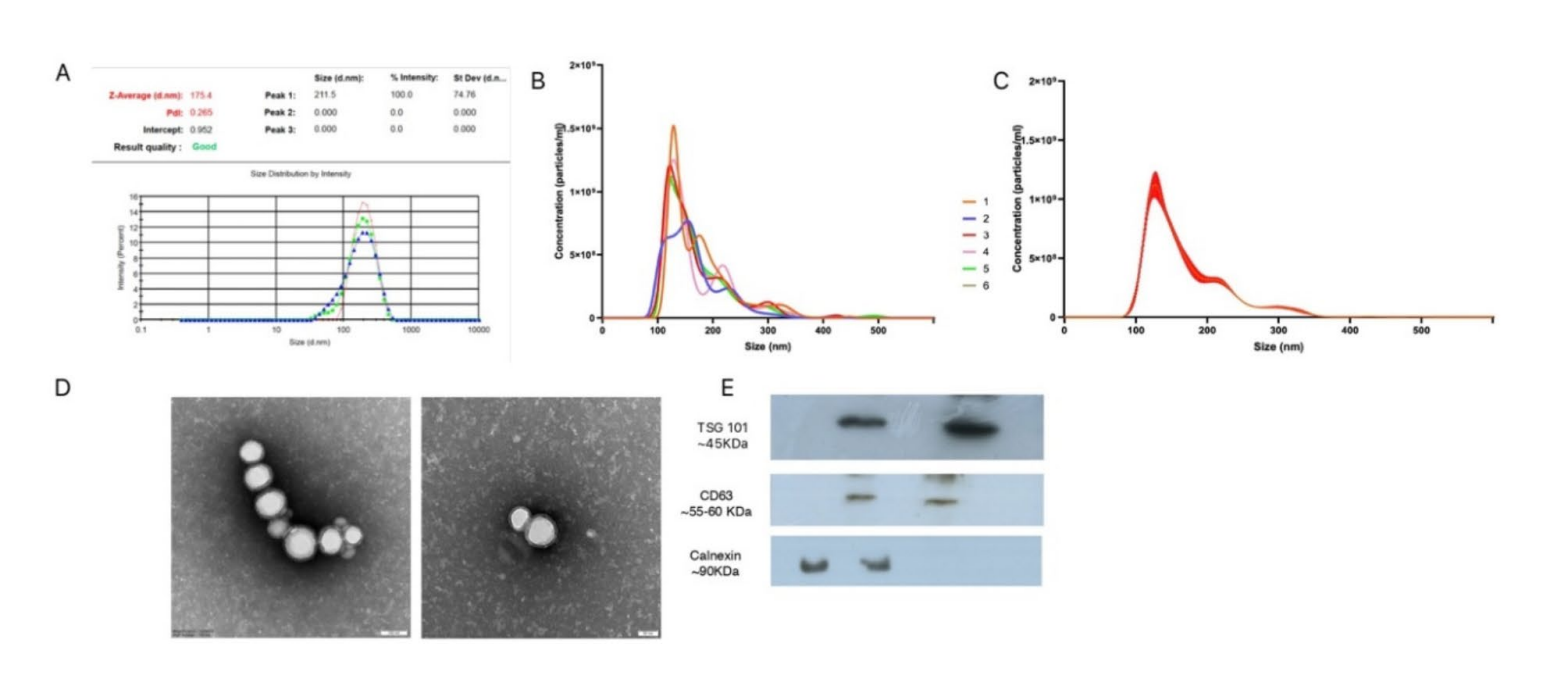
Characterization of extracellular vesicles. (**A**) Intensity-based size distribution of seminal plasma-derived EVs, as assessed by the Zetasizer nano zs particle sizer. Each curve shows means ± SD from three replicates in a representative experiment out of the three performed with similar results. (**B**) Nanoparticle Tracking Analysis (NTA) on the buffalo blood plasma-derived small EVs under 100 × dilutions. Finite track length adjustment (FTLA) size per concentration graph, taken as five replicates, the graph represents the averaged FTLA size per concentration (particles/mL) (**C**) Transmission Electron Microscopy (TEM). TEM image of EVs derived from Seminal plasma. EVs were negatively stained with 1% phosphotungstic acid after removing the extra moisture. (Magnification-100000X and 150000X, Scale bar—50 and 100 nm, 120 kV) (**E**) Identification of the TSG 101, CD63 EV-specific protein markers and Calnexin EV negative marker by the western blot analysis of isolated pooled EVs samples from the seminal plasma of *sahiwal* bulls. Full blot images are shown in Supplementary Fig. S3A–C.

Nanoparticle tracking analysis results indicate the average size and concentration of particles in fraction 7–8 was 169.0 nm + /- 57.1 nm and 8.82 × 10^10^ + /- 3.34 × 10^09^ particles/mL, in fraction 9–10 as 157.4 nm + /- 51.1 nm and 1.02 × 10^10^ + /- 4.75 × 10^09^ particles/mL, in fraction 11–12 as 105.4 nm + /- 36.7 nm and 4.74 × 10^10^ + /- 2.58 × 10^09^ particles/mL, and in fraction 13–14 as 167.4 nm + /- 55.6 nm and 7.79 × 10^10^ + /- 3.35 × 10^09^ particles/mL (Fig. 2 B, C).

The TEM analysis revealed a population of EVs with typical morphologies and sizes. TEM provided valuable insights into EV ultrastructure and size distribution (Fig. 2D). EVs were identified as small (30 – 150 nm) vesicles with a cup-shaped appearance and exhibited a characteristic lipid bilayer structure. These EVs displayed a heterogeneous size distribution, ranging from 30 – 170 nm.

The presence of SPEVs was further confirmed by Western blot using the EV protein markers, namely, a cluster of differentiation 63 (CD63), Tumor susceptibility gene101 (TSG101), and EV negative marker Calnexin (Fig. 2 E).

### Differential identification of ADAM7, SP10 and SPAM1 proteins in the SPEVs

**Fig. 3:**
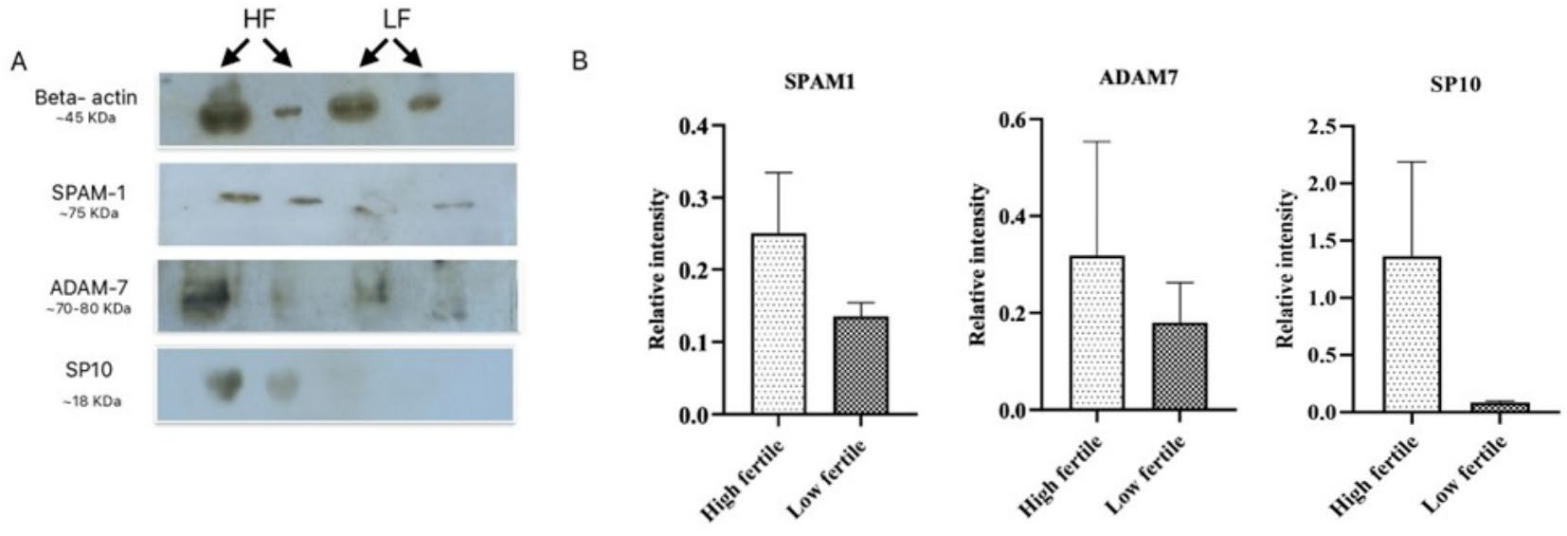
Identification of differential abundance of SPAM1, ADAM7, and SP10 using western blot. (**A**) The cropped images of the western blot show a relative abundance of SPAM1, ADAM7, and SP10 in HF and LF samples taking β-actin as a calibrator. (**B**) Histograms representing the relative intensities of SP10, ADAM7, and SPAM1 among the HF and LF samples were calculated using the blot images in the Image J software version 1.53.

We performed the western blotting of fertility-related proteins namely ADAM7, SP10, and SPAM1. Western blotting results revealed a higher abundance without significant differences of SPAM1 (p = 0.2), ADAM7 (p = 0.51), and SP10 (p = 0.16) protein in HF bull SPEVs in comparison to LF bull SPEVs (Fig. 3 A, B). The relative abundance of protein ADAM7, SP10, and SAPM1 was normalized with housekeeping protein β-actin.

### Assessment of SPEVs uptake by spermatozoa

**Fig. 4:**
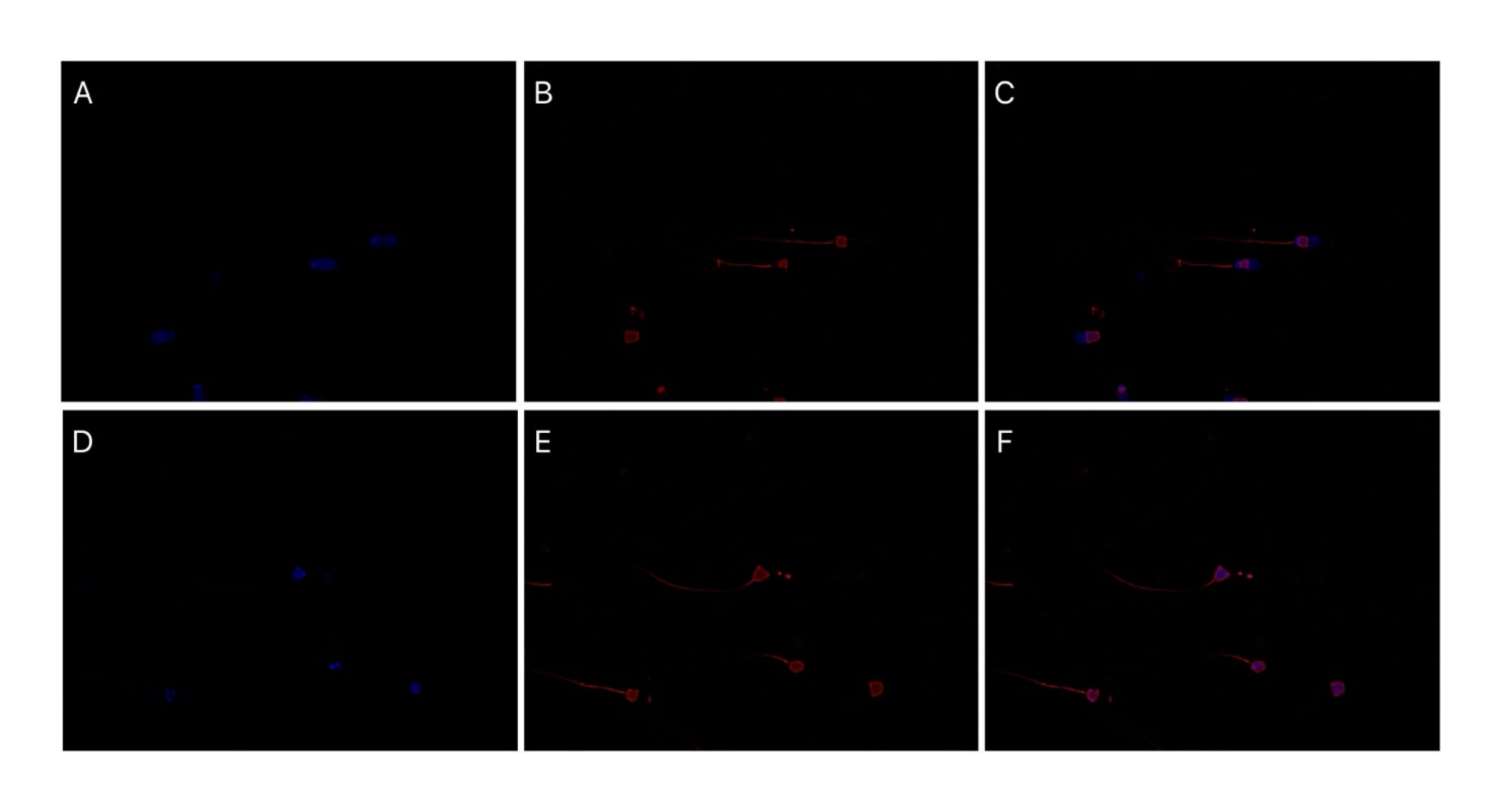
Uptake of PKH26 labeled SPEVs by the spermatozoa visualized by fluorescence microscopy. SPEVs and nucleus were stained with red and blue respectively. The images were captured at 600X magnification (**A**, **D**) spermatozoa stained with nuclei stain (DAPI-Blue). (**B**, **E**) Spermatozoa stained with lipophilic dye (PKH26 -Red). (**C**, **F**) Merged images of DAPI and PKH 26 labeled spermatozoa.

**Fig. 5:**
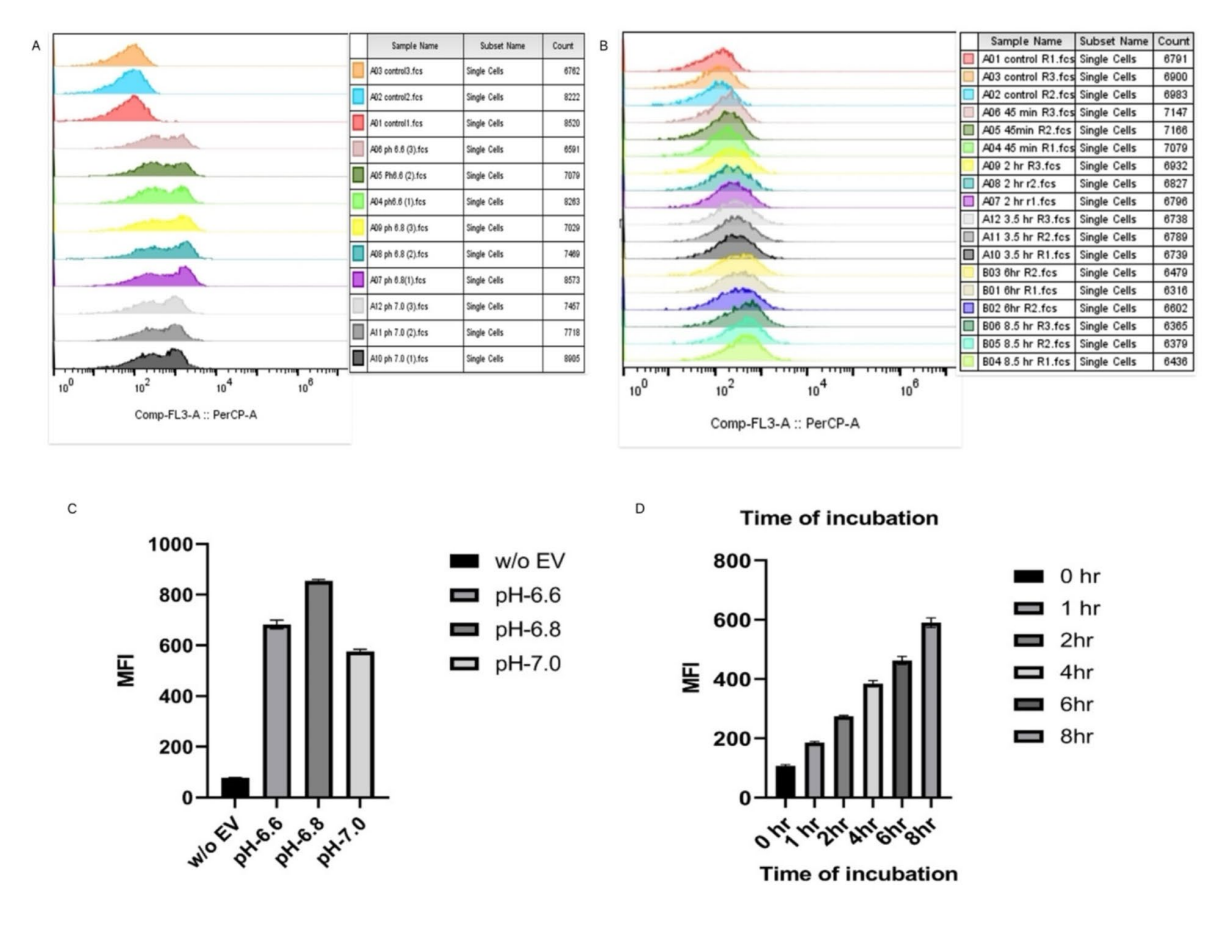
Overlay of the MFI histograms obtained by flow cytometry analysis of spermatozoa incubated with PKH26 dye (Red) labeled SPEVs at (**A**) different pH (**B**) time of incubation. Average MFI histogram of PKH26 labeled SPEVs in the spermatozoa at (**C**) different pH and (**D**) time of incubation. (n = 3).

Based on fluorescence microscopy observation, we found that SPEVs incorporation was in the sperm midpiece which appears red due to PKH-26 dye, while the heads of spermatozoa appear to be blue by DAPI. (Fig. 4). The fluorescence signal in the flow cytometer was detected on all the time points, but the maximum fluorescence signal was observed after 4 h of incubation, which was constant after this time interval (Fig. 5 A, B, C, D). The maximum fluorescence intensity signal was detected at pH 6.8. Hence, these conditions were used for all the experiments.

### Effect of HF bulls’ group derived SPEVs on in vitro sperm capacitation

**Fig. 6:**
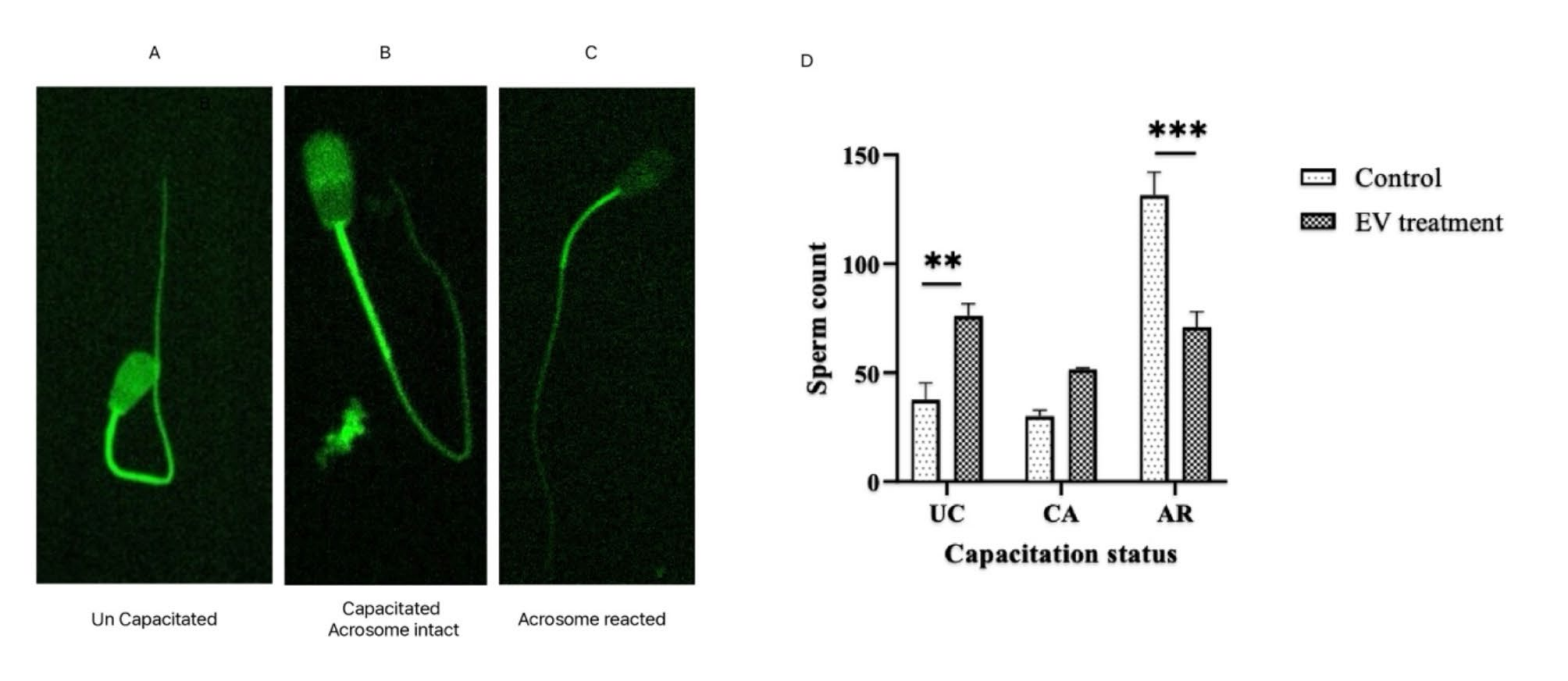
Fluorescent microscopic images of LF spermatozoa after incubation with SPEVs of HF *Sahiwal* Bulls and control. (**A**) live Un-capacitated pattern (**F**, bright green fluorescence distributed uniformly over entire sperm head, with or without stronger fluorescent line at the equatorial segment), (**B**) live capacitated pattern (**B**, green fluorescence over the acrosome region and a dark post acrosome), (**C**) live acrosome-reacted pattern (AR, live acrosome-reacted pattern, no fluorescence above the head) (**D**) Histogram representing the capacitation status of the LF spermatozoa upon incubation with HF SPEVs.

To analyze the effect of SPEVs on in vitro sperm capacitation, chlortetracycline (CTC) staining was used to determine the fluorescent patterns of sperm in the LF bulls supplemented with SPEVs derived from HF bulls. CTC was employed to observe alterations in intracellular calcium redistribution within the sperm head, specifically during capacitation. Capacitation status was classified as one of the following three patterns as previously described by Woo-Sung Kwon and co-workers^28^, live non-capacitated pattern (F, bright green fluorescence distributed uniformly over the entire sperm head, with or without stronger fluorescent line at the equatorial segment), live capacitated pattern (B, green fluorescence over the acrosome region and a dark post acrosome), or live acrosome-reacted pattern (AR, live acrosome-reacted pattern, no fluorescence above the head) (Fig. 6 A, B, C). This classification was applied to both the control group and spermatozoa exposed to SPEVs. The percentage of CTC staining fluorescent pattern F of the spermatozoa without SPEVs was found to be lower in the control group i.e. sperm without EVs and that of pattern B and pattern AR was higher in the SPEVs incubated spermatozoa (Fig. 6 D).

### SPEVs of HF *Sahiwal* bulls reduce acrosome reaction in spermatozoa of LF bulls

**Fig. 7:**
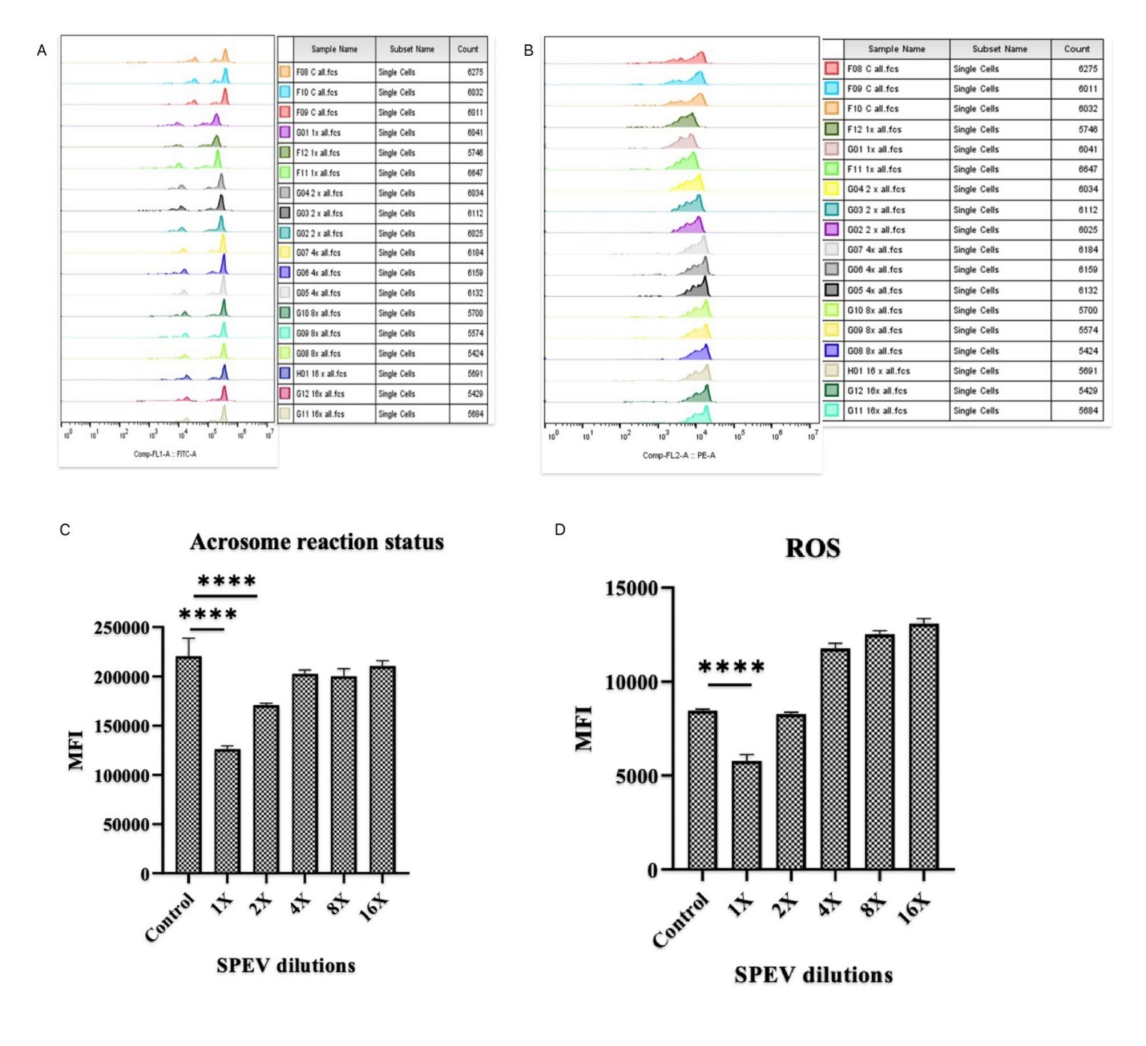
Overlay of the MFI histograms obtained by flow cytometry analysis of LF spermatozoa upon EV incubation of HF bulls at different dilutions with (**A**) FITC-PNA and (**B**) Mitosox stain. (n = 3) each group. (**C**) Average MFI histogram of acrosome-reacted sperm in an LF bull after incubation with SPEVs at different dilutions, i.e., 1X, 2X, 4X, 8X, and 16X. (**D**) Average MFI histogram of ROS production in spermatozoa of LF bulls after incubation with SPEVs at different dilutions in PBS, i.e., 1X, 2X, 4X, 8X, and 16X.

To analyze the effect of HF SPEVs on the in vitro acrosome reaction of the LF spermatozoa, we performed FITC-PNA labeling of SPEVs incubated spermatozoa to determine the acrosome status of the spermatozoa. In this context, EVs isolated from HF bulls’ seminal plasma were co-incubated with spermatozoa of LF bulls at different concentrations of SPEVs with previously described conditions. In this context, SPEVs were isolated from the HF bulls and were subsequently diluted in various dilutions such as 1X, 2X, 4X, 8X, and 16X using PBS. Ten million spermatozoa derived from LF bulls were allowed to co-incubate with SPEVs of different concentrations with 4 h of incubation at pH 6.8. The mean fluorescence intensity of treated and control spermatozoa was evaluated using one one-way ANOVA test. The flow cytometry analysis revealed that the average mean fluresence intensity (MFI) FITC-A produced in the spermatozoa upon EVs incubation was significantly reduced (p < 0.01) than the control group. However, the average MFI FITC-A was found to increase upon decreasing the SPEVs concentration (Fig. 7 A, C). A significant increase (p < 0.01) was found in diluting the SPEVs concentration to 2X. The flow cytometry analysis confirms that SPEVs of HF bulls decrease the acrosome reaction in the sperm of LF bulls. The acrosome reaction in the spermatozoa of LF bulls is positively correlated with decreasing the SPEVs concentration of SPEVs isolated from HF bulls.

### SPEVs reduce the ROS production in spermatozoa upon co-incubation

We investigated the effect of SPEVs on reactive oxygen species (ROS) production in spermatozoa upon co-incubation with SPEVs of HF bulls. EVs isolated from HF bulls were co-incubated with spermatozoa of LF bulls at different concentrations. Flow cytometry was used to quantify the ROS produced during the capacitation of buffalo sperm using the Mito SOX dye. A one-way ANOVA test evaluated the mean fluorescence intensity of treated and control spermatozoa. The flow cytometry analysis revealed that the average mean fluorescence intensity (MFI) PE-A produced in the spermatozoa upon EVs incubation was significantly reduced (p < 0.01) than the control group (Fig. 7 B, D). However, the average MFI PE-A increased upon decreasing the SPEV concentration. The flow cytometry analysis confirms the lower generation of ROS in the LF spermatozoa after treatment with SPEVs of HF bulls compared to the sperm without EVs treatment. The ROS production was found to be positively correlated with decreasing the SPEVs concentration.

## Discussion

Spermatozoa undergo several membrane modifications in the male reproductive system, these subtle modifications are mediated by the uptake of SPEVs. The SPEVs in the epididymis and prostate glands interact with the sperm and transfer their cargo to the sperm during the transit through the male reproductive tract, leading to several membrane modifications and gain of function to the spermatozoa^15,29–31^. Epididymosomes are the types of SPEVs, secreted into the reproductive tract and are released from epididymal epithelium cells. Epididymosomes interact with sperm as they pass through the epididymis after being released into the epididymal fluid^10,32^. Several molecules such as SPAM1, ADAM7, and SP10 have been reported to be transferred to the spermatozoa through the epididymosomes^29,30,33,34^. The interaction of sperm and seminal fluid EVs occurring after ejaculation indicates that the majority of their association and cargo transfer occurs in the lower female reproductive tract^35^. Interaction between sperm and SPEVs favors a slightly acidic pH, an environment like the female reproductive tract as reported in equine.

The present work was undertaken to explain whether the SPEVs from the bulls of contrasting fertility differ in their fertility-related protein abundance and to assess whether the uptake of HF SPEVs modulates the functional characteristics of LF bull spermatozoa. To prove this, we hypothesized, that a differential abundance of SPEVs protein content would affect intercellular communication between buffalo spermatozoa and the SPEVs repertoires thus augmenting sperm functions. In the current study, we have demonstrated that SPEVs of HF and LF bulls possess a different abundance of fertility-associated proteins such as SPAM1, ADAM7, and SP10. The uptake of SPEVs derived from HF bulls can improve sperm function parameters such as capacitation, acrosome reaction, and reduce the ROS production in LF bull spermatozoa.

Isolation of SPEVs was performed by SEC as described by Boeing et al.^36^. However, there are still no standardized methods to isolate the pure EVs population, as many approaches do not guarantee proper EVs purification, free of non-EV molecules and proteins. Currently, the SEC is one of the most promising techniques for purifying SPEVs from any biological fluid since it eliminates co-isolation of pollutants while maintaining adaptability. The SEC-based approach emerged as a promising technique because it minimally alters the SPEV size and characteristics when compared to other techniques such as polyethylene glycol (PEG)-based and PROSPR (protein organic solvent precipitation) and isolates the pure EVs population with a low level of contaminating soluble proteins, lipoprotein, HDL, cholesterol, etc.^37^. Therefore, we measured the size of SPEVs in each SEC fraction, to identify the appropriate SEC fractions carrying the EVs. We quantified the amount of protein in each SEC fraction and identified fractions having the majority of SPEVs with a lesser amount of free (non-encapsulated) protein followed by the fractions containing the bulk of soluble plasma proteins (Supplementary Table 1). To maintain the purity of isolated SPEVs, we chose fractions 9–12 for further analysis as these were devoid of any free-form protein. To evaluate the purity of SPEVs of contrasting fertility bulls, multiple methods of characterization were employed, on the isolated SPEVs fraction 7–12 by transmission electron microscopy (TEM), Nano sight, and western blot. Our results confirm that most of the SPEVs were within the diameter range of 50-150 nm. The TEM analysis allowed the visualization of round cup-shaped vesicles with lipid bilayer structures with diameters varying between 30-150 nm. The purity of the SPEVs population obtained by SEC was confirmed with western blot analysis by utilizing EVs markers; tumor susceptibility gene 101 (TSG 101), CD63, and Calnexin (EVs negative marker); our data concurred with the criteria for defining EVs^38^.

Further, we identified the relative abundance of fertility-associated proteins such as SPAM1, SP10, and ADAM7 in SPEVs of HF bulls in comparison to the LF bulls. Western blot-based densitometric analysis revealed a higher abundance of fertility-related proteins in the SPEVs of HF *Sahiwal* bulls. In this study, a higher abundance of fertility-associated protein SPAM1, ADAM7, and SP10 was observed in the highly fertile bulls in comparison to the bulls of low fertility status. SPAM1 plays a vital role during fertilization such as hyaluronidase enzyme activity to penetrate the hyaluronic acid-rich extracellular matrix of the cumulus cells surrounding the oocyte, ZP binding and Ca^2+^ signaling-associated acrosomal exocytosis^39^. ADAM7 protein is important for sperm cell maturation, this protein is significantly expressed in the epididymis. ADAM7 is secreted by epididymis cells into the epididymal lumen and gets transferred to the maturing sperm’s surface^26^. SP10 is an acrosomal matrix protein that plays a critical role in sperm-oocyte interaction during fertilization^40^. This protein has been reported to be evolutionarily conserved among mammals. This protein is commonly used as a male fertility indicator^41^. Altogether, the evidence of this study convincingly supports that SPEVs of HF *Sahiwal* cattle bulls possess a superior level of key fertility-associated proteins.

Following this, we wanted to know whether SPEVs of an HF *Sahiwal* bull can be uptake by the spermatozoa of an LF bull in an in vitro condition. To prove this, the SPEVs incorporation into spermatozoa was assessed by labeling the SPEVs with PKH-26 dye followed by fluorescence microscopy and flow cytometry. The mean fluorescence intensity as calculated by the flow cytometry revealed the maximum SPEVs uptake occurred at pH 6.8 and 4 h of incubation time, on increasing the incubation time a saturation in the Mean fluorescence intensity was observed. Our results indicate that cattle sperm support the uptake of SPEVs optimally at specific acidic pH and timely manner. Our finding is in concurrence with the previous study wherein the fusion of SPEVs with ejaculated sperm was reported at a slightly acidic pH, possibly like the female reproductive tract^42^.

The question that arises here is whether the fusion of SPEVs of HF bulls with spermatozoa of LF bulls improves the functional characteristics of LF bull spermatozoa. To determine the effect of the assimilation of SPEVs of HF bulls with the spermatozoa of LF bulls, we assessed several sperm functional tests such as the acrosome reactions, capacitation, and ROS production by LF bull spermatozoa. Sperm capacitation and acrosome reaction are the two vital functions that allow the sperm to reach the site of fertilization and penetrate the ZP to fuse with the oocyte membrane. As the spermatozoa move out of the ejaculate passes through the cervical mucous they undergo several biochemical changes collectively called capacitation^43^. At the time of fertilization, the capacitated spermatozoa penetrate the cumulus oophorous of the ovum, followed by binding to the ZP with an intact plasma membrane. After binding to the ZP, the spermatozoa undergo a physiochemical process called acrosome reaction^43,44^. In this study, we report a significant decrease in the acrosome reaction and capacitation in the spermatozoa of LF bulls upon uptake of SPEVs of HF bulls. This can be explained due to the reason that assimilation of SPEVs of HF bull origin along with delivery of abundant protein cargo including SPAM, SP10, and ADAM1 to the LF spermatozoa at a favorable pH may inhibit the premature acrosome reaction and capacitation in the spermatozoa of the LF bulls.

A certain level of ROS is required for the maturation of spermatozoa, acrosome reaction, capacitation, hyperactivation, and sperm-oocyte fusion^45^. The increased ROS production with reduced antioxidants leads to the redox imbalance, thereby reducing sperm motility and causing sperm DNA damage. ROS are deleterious to spermatozoa due to the large amount of unsaturated fatty acid found in the cell membrane of spermatozoa. The ROS production promotes lipid peroxidation, loss of membrane integrity, reduced sperm motility, structural DNA damage, and apoptosis in the spermatozoa^46^. We observed a reduction in ROS production upon incubating the LF spermatozoa with the SPEVs derived from HF bulls, which has been demonstrated to be highly associated with improving the LF spermatozoa. However, additional evidence for the fusion of individual SPEVs proteins with LF bull spermatozoa may be needed to confirm this assumption.

## Conclusion

The current study provides clear evidence that ejaculated mature bovine sperm are receptive to the uptake of the SPEVs in vitro conditions. Maximum uptake of SPEVs by spermatozoa occurs at a slightly acidic pH like vaginal pH. The SPEVs of HF bulls carry a high abundance of fertility-related proteins and this proves that SPEVs carry fertility-supporting protein cargo which can improve the spermatozoa function of the LF bulls. The molecular content particularly the protein cargo present in the SPEVs of HF bulls may augment the functional characteristics of the LF bull spermatozoa. Our findings indicate a possible strategy to improve the breeding outcome of LF bulls of high genetic merits. The limitation of the current study is that SPEVs of HF bulls may possess numerous proteins and other metabolomes that may elicit inhibitory effects on sperm functions. Hence, further explanations are warranted in this direction by a study with a pure population of individual protein to prove the effect and efficacy of uptake candidate protein as well as to understand the complex mechanisms triggered due to the uptake of protein through SPEVs to the bull spermatozoa in *vitro* conditions.

## Materials and methods

All reagents were purchased from Sigma-Aldrich, while test tubes and culture plates were purchased from Thermofisher. Raw semen samples of *Sahiwal* bulls (*Bos indicus*) were obtained from an Animal Breeding Research Centre (National Dairy Research Institute, Karnal, Haryana, India).

### Selection of bulls for SPEVs preparation

Over thirty standing *Sahiwal* bulls, each with more than 80 insemination records, were analyzed for their fertility classification (Fig. 1). These bulls were raised under a regular feeding and management plan. The selected bulls were for the progeny testing program based on evaluations of their breeding soundness and semen quality, which included factors like semen volume, sperm count, viability, and progressive motility. The selected bulls were managed according to the standard feeding and management protocols of Artificial Breeding Research Center (ABRC), NDRI, Karnal, Haryana, India.

The data on conception rates (CRs) was obtained from the ABRC, NDRI, Karnal, Haryana. The CRs of the 31 bulls were considered for normality using the Shapiro–Wilk test, and the data was fit into a normal distribution (p = 0.059, confirming the null hypothesis). The mean CR was calculated to be 44.49% with a standard deviation (SD) of 7.89%. Consequently, the bulls with CRs falling between 49.12% and 39.34% were categorized as averagely fertile (Fig. 1). For this study, ten *Sahiwal* bulls (*Bos indicus*, n = 10) were selected, among them we were able to collect semen samples from four high fertility bulls (HF, n = 4, CR between 53% and 59.59%) and three low fertility bulls (LF, n = 3, CR between 29.15% and 33.3%). The chosen bulls had CRs above and below the Mean ± 1 S.D (HF > 52.94% and LF < 38.01%), as referenced in previous studies^47,48^.

### Seminal plasma collection and sample pre-processing:

Freshly ejaculated normozoospermia semen samples (mass motility ≥ 3, aged between 3 and 5 years) were collected from mature *Sahiwal* bulls (of proven fertility) using the artificial vagina at the Artificial Breeding Research Centre (NDRI, Karnal, India). Each ejaculate was collected in a 15 mL centrifuge tube and immediately transported to the laboratory at 37 °C. The semen sample was centrifuged at 3000 × g for 15 min and the supernatant was carefully collected to isolate the seminal plasma from the spermatozoa. We collected approximately 0.5 mL of semen from each ejaculate of the bull, with a minimum of three ejaculates per bull being used for the study. The seminal plasma from these collections was then pooled for further analysis. The seminal plasma was stored at—-80 °C for further use. At the time of processing, the sample was thawed and centrifuged at 10,000 g for 30 min to pellet down the debris. The supernatant was transferred to a fresh 1.5 mL tube for further investigation.

### Size exclusion chromatography for isolation of SPEVs

Isolation of vesicles by SEC was performed as described by Boïng et al.^36^ with minor modifications. For SEC, Sepharose CL-2B (10 mL Sigma Aldrich, St. Louis, MO, USA) was stacked in a 20 mL syringe column (BioRad), washed, and equilibrated with PBS + 0.32% trisodium citrate (0.22 µm). Less than 1 mL of seminal plasma was loaded onto the column and fraction collection (0.5 mL per fraction and a total of 30 fractions were collected) started immediately using PBS + 0.32% TCS as elution buffer Click or tap here to enter text..

### Characterization of isolated SPEVs

The isolated SPEVs were characterized using various previously reported methods as per MISEV2018 guidelines. Three consecutive fractions were pooled together in a single pool for the characterization of isolated EVs by Western blot, DLS, Nanoparticle tracking assay (NTA), and Transmission electron microscopy (TEM).

### Nano-tracking analysis of SPEVs

To assess the concentration and size of nanoparticles in fractions 7–14, a NTA was conducted. All particle tracking analyses were performed using an NS300 unit (Malvern) equipped with a 488 nm laser and a 500 nm long-pass filter for fluorescence detection. All samples were diluted in 10:1000 with 1 X PBS to be counted using NTA. All counts were performed in replicates of 5 for each sample, collecting 30–60-s videos with a minimum of 200 valid tracks recorded per video (minimum of 1000 valid tracks recorded per sample). The NTA analysis was performed by combining two consecutive fractions, namely 7–8, 9–10, 11–12, and 13–14 (Supplementary Table 1). Nanosight 3.0 software was used for all analyses, using standard settings. The camera level for each sample was manually adjusted to achieve optimal visualization of particles. For all experiments, the camera level setting ranged from 12–14 samples analyzed in light scatter mode (LSM) and from 15–16 samples analyzed in fluorescence mode (FM). The detection threshold (DT) was set for maximum sensitivity with a minimum of background noise, with the level ranging from 5–7 samples analyzed in LSM and from 3–4 samples analyzed in FM. The sample infusion pump was set to a constant flow rate of 5 μL/min. To minimize variability, all camera and detection threshold settings were kept the same for each mode when performing multiple experiments on a single sample source.

### Determination of SPEVs size by dynamic light scattering

The size of exosomes was evaluated using a Zetasizer Nano ZS system (Malvern Instruments, Malvern, UK) involving the DLS technique. This technique is based on analysing the velocity distribution of particle movement by measuring the dynamic fluctuations of scattered light intensity at a fixed angle caused by the Brownian motion of the particle and the particle’s hydrodynamic radius, or considered diameter, is then calculated with the Stokes–Einstein equation. After exosome isolation with the SEC, the 0.5 mL three fractions were pooled together and analyzed. purified SPEVs were diluted in a ratio of 1:20 with the filtered 1X PBS followed by sonication in an ultrasonic water bath for 1 min. The whole volume was quickly put in a disposable cuvette for size measurements to avoid aggregation of the exosomes. Three independent measurements were performed for each sample and averaged by the software for the analysis.

### Transmission electron microscopy

SPEVs were diluted 1:100 in 1X PBS and fixed with an equal volume of 2% paraformaldehyde (in PBS) for 1 h at 4 °C. Carbon Formvar film-coated 300-mesh TEM grids were glow-discharged before use. The fixed EVs were then applied to the grids and incubated at room temperature for 15 min. After washing with PBS, the sample was fixed with 1% glutaraldehyde for 5 min onto the grid. The grids were then rinsed with distilled water and stained with 1% phosphotungstic acid for 45 s. Excess stain was wicked off using Whatman filter paper, and the grids were allowed to air dry. TEM imaging was conducted using a JEM1400 FLASH (JEOL USA Inc., Peabody, MA) at 120 kV, with images captured at 250,000 × magnification by an Olympus sCMOS camera. This analysis was performed at the Advanced Technology Platform Centre (ATPC) facility of the Regional Centre for Biotechnology, Faridabad, Haryana, India.

### Western blot analysis of SPEVs protein

The SPEVs protein was isolated by incubating the isolated SPEVs in RIPA buffer (Sigma-Aldrich) for 15 min at room temperature, followed by sonication. The lysed protein from all pools was precipitated using Acetone (1:5). The protein concentration was determined using a Bradford protein assay according to the manufacturer’s instructions (Pierce, Rockford, IL). The absorbance was measured at 595 nm on an infinite M Nano Tecan plate reader. The SPEVs protein was isolated from 4 HF and 3 LF bulls and pooled together in two groups of HF and LF SPEVs proteins to remove any biological variation. Approximately, 20 μg of protein was loaded per well into SDS-PAGE gel, and proteins were separated by 12% SDS polyacrylamide gel electrophoresis using a mini gel tank electrophoresis system (Invitrogen Life Technologies) and then transferred to Immobilon-FL polyvinylidene difluoride membranes (Millipore, Billerica, MA, USA). The membrane containing the transferred protein was probed with primary mouse polyclonal anti-TSG-101 (1:1000 SC-7964, Santa Cruz Biotechnology, USA) primary goat anti-CD63 (1:3000, STJ140029, St. Johns Laboratory, London, United Kingdom), primary Anti-Calnexin (CNX) Monoclonal Antibody (CAA280Hu22, Cloud Clone Corp, USA), primary polyclonal antibody against ‘A Disintegrin and Metalloprotease protein’ (ADAM7) (1:5000, PA5-44,080, Invitrogen), primary anti-Synaptobrevin (SP10) monoclonal antibody (ab18013, abcam), anti SPAM1 mouse monoclonal antibody (ab50693, Abcam, USA) anti β-actin antibody (1:20,000, AM4302, Thermo Fisher Scientific). Membranes were washed in Tris-buffered saline (TBS- pH 7.6) and incubated for 2 h in TBST (TBS with Tween-20) containing 5% Bovine Serum Albumin (BSA) along with horseradish peroxidase-conjugated anti-mouse secondary antibody for TSG101 (1:10,000, SC-516102, Santa Cruz Biotechnology), anti-goat secondary antibody for CD-63 (1:20,000, STJ-99512, St. Johns laboratory), anti-mouse secondary antibody for calnexin (1:10,000, A9044, Sigma) anti-rabbit secondary antibody for ADAM7 (1:30,000 Sigma, 12,348), anti-mouse secondary antibody for SP10 (1:20,000, A9044, Sigma), anti-mouse secondary Ab for SPAM1 and β-actin (1:30,000, 1:25,000, respectively A9044, Sigma). The Substrate Pierce ECL Western Blotting kit (32,106) was utilized in the next step for chemiluminescence, and subsequently, the membranes were exposed to X-ray film for 1–5 min before visualization.

### Fluorescence labelling of SPEVs

The isolated SPEVs were stained with PKH26 dye using PKH26 Red Fluorescent Cell Linker Kits for General Cell Membrane labeling (Sigma-Aldrich). Before staining, 2 µL of PKH26 dye in 498 µL of diluent C was incubated in an ultrasonic water bath at 37 °C for 15 min. SEC fractions 7–12 were collected and pooled together followed by concentrating the pooled SPEVs samples to a volume of 100 µL. The concentrated SPEVs diluted in 100 µL of diluent C (provided by the manufacturer) were pipetted on the PKH26 solution and incubated for 5 min at 37 °C. Excess staining was stopped by adding an equal amount of 1% BSA dissolved in 1X PBS. SEC of the labeled SPEVs sample was carried out as previously described to remove the unbound PKH 26 dye. We loaded ~ 1 mL of the stained SPEVs sample on the CL-2b size exclusion chromatography column. We harvested 20 fractions of 500 µL each and then pooled the 7–12 fractions as previously described by Kastresana et al.^49^, later analyzed by differential light scattering. These experiments were repeated at least three times with similar results.

### Spermatozoa incubation with labelled SPEVs

To assess the uptake of SPEVs by the LF bull spermatozoa, we labeled purified extracellular vesicles with the fluorescin-based lipophilic PKH-26 and then co-incubated with *Sahiwal* bull (n = 3) spermatozoa (1 × 10^6^) for 2 h at 37 °C in the dark followed by sperm visualization under fluorescence microscopy. We further investigated the optimum time of co-incubation required in our model, the SPEVs stained with PKH26 dye were incubated with spermatozoa in a time-dependent manner, i.e. 1, 2, 3, 4, 6, and 8 h in the SP-TALP medium, and analyzed by flow cytometry. We investigated the optimum pH for the SPEVs uptake by the sperm. The labeled SPEVs were incubated with spermatozoa (1 × 10^6^) in an SP-TALP medium on varying pHs 6.6, 6.8, and 7.0 and incubated at 37 °C for 4 h in the dark followed by flow cytometry analysis.

### Evaluation of in vitro capacitation using CTC assay after incubation of spermatozoa with SPEVs

The in vitro capacitation status of the LF bull spermatozoa was observed after incubating the spermatozoa with the SPEVs derived from the HF bulls. Sperm were incubated with SPEVs in the SP-TALP media for 6 h in a 5% CO_2_ incubator (Innova-Co 170; New Brunswick Scientific, Edison, NJ, USA), at 37 °C after washing twice in the SP-TALP media. The sperm capacitation was confirmed by CTC staining, a fluorescent chelate probe of Ca^2+^, by the method described by Woo-Sung Kwon; Saraf et al.^28,50^ with some modifications. Working CTC solution was prepared by dissolving 5 mM cysteine (6.06 mg per 10 mL), 130 mM NaCl (75.97 mg per 10 mL), 20 mM Tris–HCl (31.53 mg per 10 mL), and 750 mM CTC–HCl (3.87 mg per 10 mL) into PBS. Equal amounts of the prepared stain solution (20 mL) and sperm suspension (10 million) were added to a 0.5 mL microcentrifuge tube, and the tube was then incubated for 15 min at 37 °C in the dark. The sperm solution was then given an additional 2 min of incubation after adding 3 mL of 4% PFA. Spermatozoa were rinsed with sperm-TALP after incubation. Equal amounts of the stain solution (20 mL, manually prepared) and sperm suspension (10 million) were added to a 0.5 mL microcentrifuge tube and were then incubated for 15 min at 37 °C in the dark. The sperm solution was then given an additional 2 min of incubation after adding 3 mL of 4% PFA. The spermatozoa were washed with 200 μL of SP-TALP and centrifuged at 800 × g for 3 min, for the removal of excess stains. The pelleted spermatozoa were used to create a thin smear, a few drops of 1,4-Diazabicyclo-octane (DABCO 33-LV) (Sigma Aldrich, St. Louis, MO, USA) mounting media were added, and the smear was viewed at 600X using an Olympus BX-51 fluorescent microscope with a UV filter (450 nm emission and 350 nm excitation). Each sample was analyzed twice, with at least 200 spermatozoa per slide.

### Acrosome integrity test in spermatozoa after SPEVs uptake by LF bull sperm

To determine the acrosome status of spermatozoa after incubating them with SPEVs derived from HF bulls at different concentrations as previously described, fluorescein isothiocyanate conjugated peanut agglutinin (FITC-PNA) (λex 494 nm and λem at 517 nm) was added in conjugation with propidium iodide (PI)^51^. In a nutshell, 10 million spermatozoa were treated with 5 μL of FITC-PNA (25 μg/mL) and were then incubated at 37 °C for 15 min in the dark. After incubation, 1 μL of PI was added and incubation was carried out for 2 min. Approximately, 200 μL 1X SP-TALP was added to the samples and then centrifuged at 800 g for 3 min to remove the extra stain. After removing the supernatant, the spermatozoa were resuspended in 100 μL 1X SP -TALP and analyzed using a flow cytometer (BD accuri) by considering 25,000 events. The flow cytometry results were analyzed using FlowJo v10.8 Software (RRID:SCR_008520; BD Life Sciences).

### Evaluation of ROS production using MitoSOX assay after incubation of spermatozoa with SPEVs

The ROS production in the LF spermatozoa was observed after incubating the spermatozoa with the SPEVs derived from the HF bulls. Sperm (0.2 × 10^6^) were incubated with SPEVs in the SP-TALP media for 4 h in a 5% CO_2_ incubator (Innova-Co 170; New Brunswick Scientific, Edison, NJ, USA), at 37 °C after washing twice in the SP-TALP media. Approximately, 200 µL of 1X SP-TALP was added to the samples and then centrifuged at 700 g for 5 min. To determine the ROS production in SPEVs incubated spermatozoa, working MitoSOX red (5 μM) was added in each tube and incubated for 30 min. 200 µL of 1X SP-TALP was again added to the samples and then centrifuged at 700 g for 5 min. The acquired sample was resuspended in 100 µL 1X SP-TALP and reading was taken in flow cytometry as mentioned above.

### Statistical analysis

The sperm functional parameters and protein quantification through Image J software version 1.53 (RRID: SCR_003070) were subjected to statistical analysis at a 95% confidence level (p < 0.05) using the unpaired student’s *t*-test. The values have been expressed as mean ± SEM (Standard error mean). All graphing and statistical analyses were carried out using GraphPad Prism (GraphPad Prism, RRID: SCR_002798).

## Electronic supplementary material

Below is the link to the electronic supplementary material.

Supplementary Material 1

## Data Availability

The datasets supporting the conclusions of this article are included within the article and its supporting information.
