# Supplementary material for: Extracellular vesicles in seminal plasma of Sahiwal cattle bulls carry a differential abundance of sperm fertility-associated proteins for augmenting the functional quality of low-fertile bull spermatozoa
Source: Sci Rep. 2025 Jan 28;15:3587. doi: 10.1038/s41598-025-87998-2 (PMC11775099; doi:10.1038/s41598-025-87998-2)
Supplement: Supplementary file 1 — Supplementary Material 1 [file 41598_2025_87998_MOESM1_ESM.pdf]

Supplementary Information

Protein quantification of SPEVs in SEC fractions

Since the SPEVs containing fractions might be contaminated with the soluble protein, therefore we quantified the protein concentration in all 10 pools. The protein concentration was determined using a standard curve of bovine serum albumin (BSA) assessed by BCA assay (Thermofisher Scientific) according to the manufacturer's instructions. The absorbance was measured at 562 nm on an Infinite® 200 NanoQuant microplate reader (Tecan).

The Bradford protein analysis was used to determine the total protein concentration of the un-lysed EV pooled fractions. Initially, up to fraction 15, a lower amount of protein was detected, which can be attributed to the encapsulation of proteins within the EVs. However, a maximum amount of protein was detected in the later fractions (from fraction 16 onwards), indicating a higher protein content as these fractions primarily contained soluble proteins. (Supplementary Figure 1)

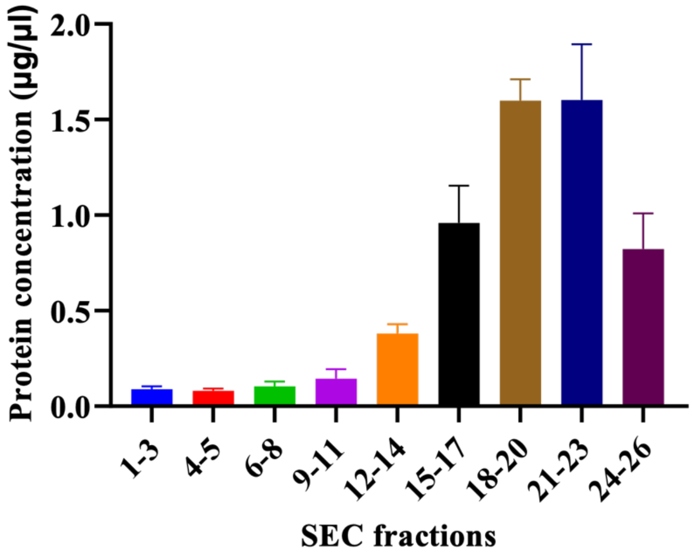

Supplementary Figure 1: Concentration of protein in the different pools of SEC fractions

TSG101

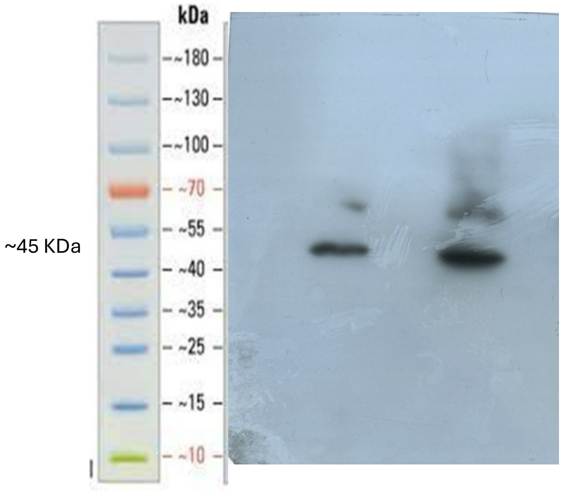

CD 63

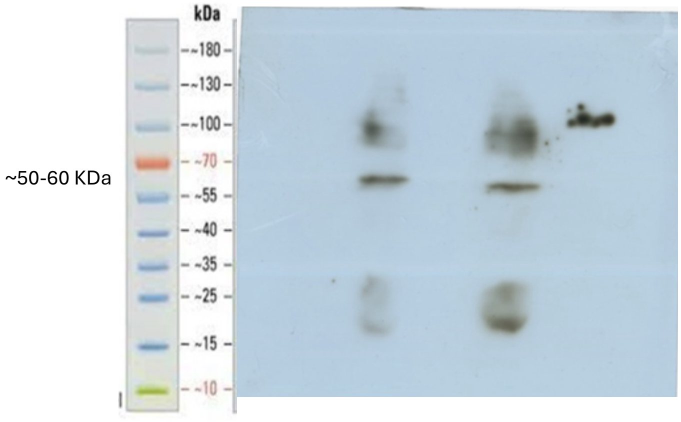

Calnexin

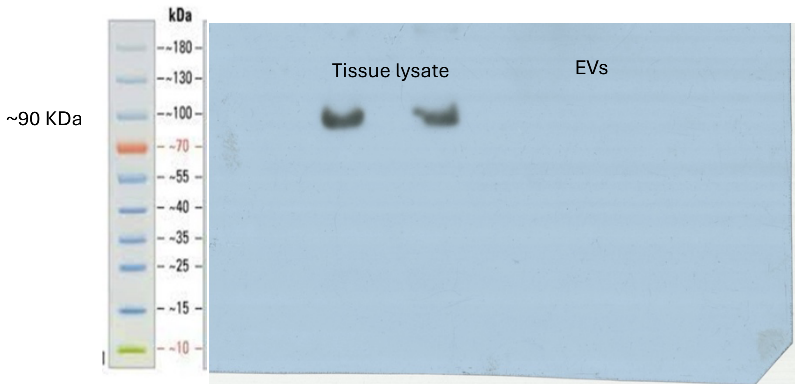

Supplementary Figure 2: Western blot (full) images of the EVs-specific markers (A) TSG101 (B) CD63 (C) Calnexin (EVs negative marker)

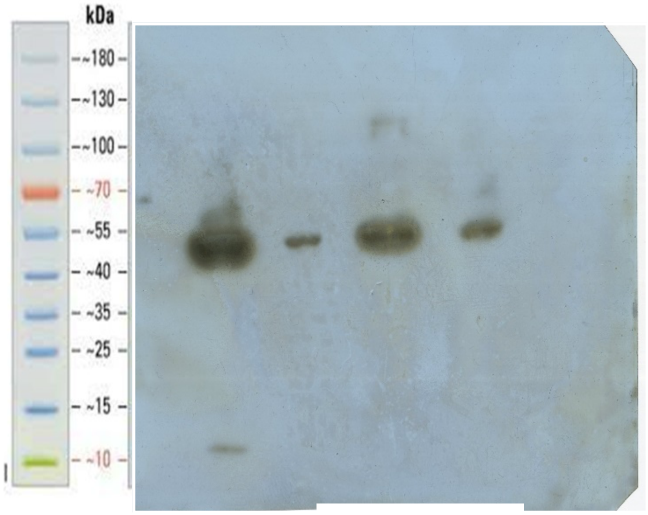

β- actin

~ 42KDa

~70-75 KDa

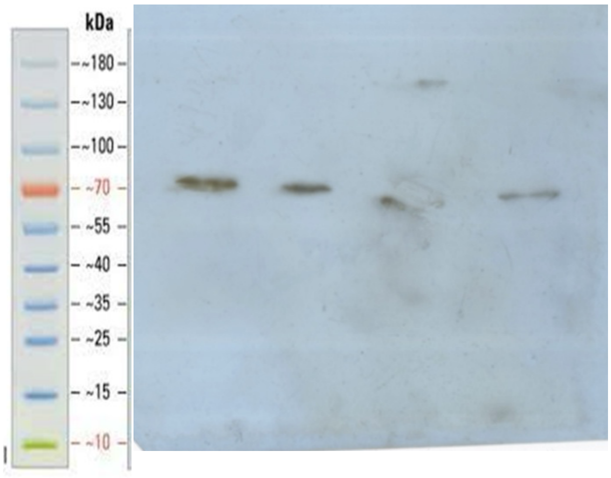

SPAM1

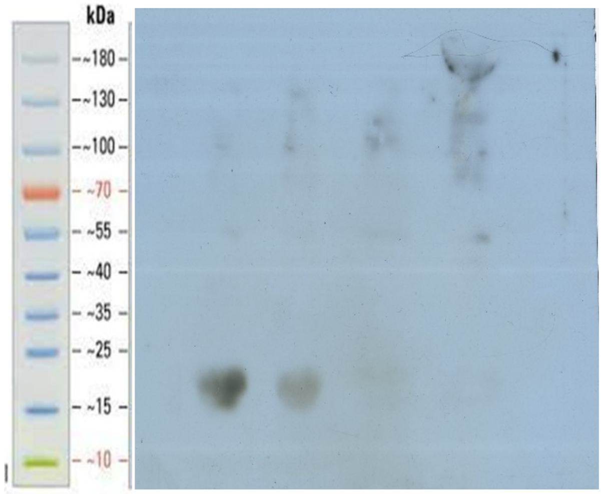

SP10

~70-80 KDa

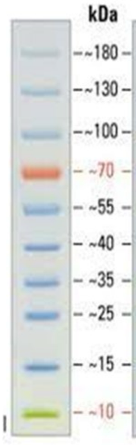

A

ADAM 7

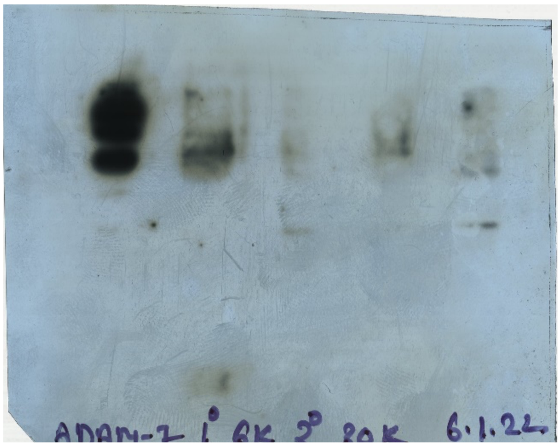

Supplementary Figure 3: Western blot (full size) images of the β actin, SPAM 1, SP10 and ADAM7.

~18 KDa

Supplementary Table 1: Size and concentration-based distributions of the SEC pooled fractions

S. No.	SEC fraction number	Mean Size	Concentration (particles/ml)
1.	7-8	168.7 +/- 2.1 nm	8.82e+10 +/- 3.34e+09
2.	9-10	157.7 +/- 1.6 nm	1.02e+11 +/- 4.75e+09
3.	11-12	105.8 +/- 2.3 nm	4.74e+10 +/- 2.58e+09
4.	13-14	167.5 +/- 2.5 nm	7.79e+10 +/- 3.35e+09
